# Emergency and disaster preparedness in the zonal hospitals in the Somali Regional State, Ethiopia: a cross-sectional study

**DOI:** 10.1007/s43999-026-00098-0

**Published:** 2026-09-10

**Authors:** Bashir Farah, Milena Pavlova, Wim Groot

**Affiliations:** 1 Department of Health Services Research; CAPHRI, Maastricht University Medical Center, Maastricht University, P.O. Box 616, Maastricht, 6200 MD The Netherlands; 2 Administration and Development of Jigjiga University, Jigjiga, Somali Regional State Ethiopia; 3 Department of Health Services Research, Faculty of Health, Medicine and Life Sciences, School of Care and Public Health Research Institute, CAPHRI, Maastricht University Medical Center, Maastricht University, PO Box 616, Maastricht, 6200 MD The Netherlands

**Keywords:** Emergency preparedness, Disaster management, Hospital safety index, Health system resilience, Somali region, Ethiopia

## Abstract

**Background:**

Hospital preparedness is essential for effective response to disasters and emergencies. Nevertheless, empirical evidence about hospital disaster preparedness is scarce in most low- and middle-income contexts, including Ethiopia. This study evaluated the degree of emergency and disaster preparedness in general public hospitals in the Somali Regional State of Ethiopia.

**Methods:**

A facility-based cross-sectional survey was performed from May to July 2023 in four general public hospitals located in the Somali Region. Data was gathered utilizing Module 4 of the WHO Hospital Safety Index checklist, which evaluates emergency and disaster management preparedness across 40 indicators.

**Results:**

The hospitals assessed demonstrated insufficient preparedness, attaining a moderate score of 45%. The preparedness for emergency and disaster response and recovery was the lowest, at 41.3%, among the seven evaluated domains. Substantial weaknesses were present in essential areas like emergency response planning, logistical readiness, financial administration, and communication systems. Hospitals lacked systematic communication strategies with the public and media during emergencies and failed to keep an inventory of external stakeholders for coordinated response initiatives.

**Conclusion:**

The findings demonstrate that general hospitals in the Somali Region are moderately equipped to manage emergencies and disasters effectively. Improving hospital systems for disaster management, enhancing communication and coordination, and preparing for logistical and financial issues are imperative. To make the region’s healthcare system more resilient and ready for emergencies, hospital administrators, regional health authorities, and the Federal Ministry of Health must work together.

## Highlights

General Public hospitals in the Somali Region have moderate resilience to disasters, ranking emergency and disaster preparedness response and recovery as their least prepared area.

The coordination of emergency and disaster management activities in the Somali Region hospitals is still ineffective, with a nominal structure lacking the proper functions of an incident management system and no assigned staff members.

The communication and sharing of information in the Somali Region hospitals did not meet the expected level of readiness.

No hospital plan in the Somali Region currently includes a full provision of psychosocial support to emergency victims during emergency and disaster events.

## Introduction

Disasters are serious disruptions in the functioning of a community that result in widespread human, economic, material, and environmental losses exceeding the affected community’s capacity to cope using its resources [1]. The World Health Organization (WHO) defines disasters as extensive ecological incidents needing external aid [2]. Disasters manifest worldwide, although they disproportionately affect low- and middle-income countries (LMICs) owing to their limited resources, deficient infrastructure, and scarcity of skilled health professionals [3]. Such events can cause injuries, deaths, destruction of health infrastructure, and long-term psychological and social consequences for affected populations and healthcare providers [4, 5].

Hospitals play a critical role during disasters and emergencies by providing essential medical care to affected populations. However, disasters can simultaneously damage hospital infrastructure, increase demand for care, and disrupt services, thereby limiting hospitals’ ability to respond effectively [6, 7]. Consequently, hospital disaster preparedness has become a global public health priority [8]. International agencies, including WHO and the Pan American Health Organization (PAHO), emphasize the importance of emergency planning, coordination mechanisms, trained personnel, communication systems, logistics management, and protection of critical infrastructure to ensure continuity of care during emergencies [8, 9].

In Africa, the burden of disasters continues to increase, while hospital preparedness remains limited in many countries. Studies conducted in Namibia, Tanzania, and other African contexts reveal that disaster preparedness in healthcare institutions is still in its early stages [10–13]. These gaps are particularly concerning in regions frequently affected by climate-related disasters, disease outbreaks, and humanitarian crises, where healthcare systems often operate under resource constraints [14].

Ethiopia has a significant risk of the occurrence of disasters and emergencies due to recurrent droughts, floods, epidemics, and emergencies from conflicts [15, 16]. Despite an increase in the country’s health facilities over the past few decades, various studies from different regions of the country still indicate the poor disaster preparedness of hospitals and limited emergency response capacity, which was clearly witnessed during the COVID-19 pandemic [17–20]. The country has more than 350 hospitals with over 40,000 beds across nationwide. However, this does not mean that hospital care is proportionally available. In the Somali Region, where disaster and humanitarian emergencies are high-risk, only one referral hospital, four general hospitals and thirteen primary hospitals serve the scattered population with some 2,500 beds. This is exacerbated by a shortage of health professionals and geographic challenges to accessing health facilities [21, 22]. These challenges underscore the critical importance of ensuring that hospitals in the region are adequately prepared to respond effectively to emergencies and disasters [23].

Despite the disaster-prone nature of the Somali Region, empirically verifiable studies about emergency and disaster preparedness of hospitals in the region are scant [22]. Furthermore, little is known about hospital preparedness for major elements, including coordination, emergency response planning, human resources, logistics and finance, communication, patient services and support, and evacuation. Filling these knowledge gaps is very crucial in strengthening health systems to prepare for disasters [24–30].

Thus, this study was undertaken to evaluate the extent of emergency and disaster preparedness of general public hospitals (GPHs) in the Somali Regional State of Ethiopia. In particular, the study intended to determine the level of preparedness across the major domains of hospital disaster preparedness and identify areas of concern and high priority. The research question for this study was “To what extent are the GPHs in the Somali Region prepared for disaster, and what aspects of preparedness need the most attention to improve the disaster response capability?”

## Methods

### Study design

A facility-based cross-sectional survey was conducted from May to July 2023 in selected general public hospitals in the Somali Regional State of eastern Ethiopia. Using the WHO Hospital Safety Index emergency and disaster management module [31], the study evaluated preparedness across key domains of hospital disaster management. The research was conducted in four zonal hospitals situated in the Fafan, Jarar, Korahay, and Shabelle zones, which are part of the eleven administrative zones in the region. These zones collectively serve well over three million people and are prone to recurrent droughts and seasonal flooding, underscoring the need for strong hospital emergency and disaster preparedness.

### Study setting

#### Geographic and health system context

Ethiopia has a decentralized health system organized into federal, regional, zonal, and district (woreda) administrative levels [32, 33]. Health services are delivered through a three-tier healthcare delivery system, which includes primary healthcare units (health posts, health centers, and primary hospitals), secondary-level general hospitals, and tertiary specialized hospitals. The country has expanded its health infrastructure significantly over the past two decades through the Health Extension Program and primary healthcare expansion, which have improved access to essential health services across both urban and rural areas.

### Health infrastructure

Within Ethiopia’s national health system framework, the Somali Regional Health Bureau (SRHB) oversees the regional healthcare delivery system [34–36]. The public health infrastructure in the region currently includes 18 public hospitals (13 primary hospitals, 4 general hospitals, and 1 specialized hospital), 242 health centers, and 1,596 health posts. In addition, 29 mobile health and nutrition teams provide outreach services to pastoralist and remote communities with limited access to fixed health facilities. The private health sector also contributes to service delivery and includes more than 600 private health facilities, ranging from clinics to primary hospitals [31, 37–41].

We included four government secondary-level (general) hospitals with operational emergency departments in the present study. These hospitals serve as referral facilities for primary healthcare units and play a critical role during mass casualty incidents, disease outbreaks, and climate-related emergencies. Consequently, they represent key nodes within the regional emergency and disaster response system.

### Sampling strategy and study participants

The study targeted general public hospitals located in four provinces, such as Fafan, Jarar, Korahay, and Shabelle, which represent some of the most densely populated areas of the Somali Regional State of Ethiopia. A purposive sampling strategy was employed to select hospitals providing secondary-level healthcare services, named general hospitals, as these facilities serve as referral centers for primary healthcare units and play a central role in responding to emergencies and disasters.

In the study area, five public hospitals were operational at the time. However, the regional referral hospital was intentionally excluded because it provides tertiary-level specialized services, which differ substantially from the operational scope and organizational structure of general hospitals. Consequently, four general hospitals were included in the assessment.

The selected hospitals were chosen based on several criteria, including similarity in service level, year of establishment, population coverage, representativeness of the selected zones, and their functional linkages with surrounding primary healthcare facilities, such as health centers and health posts. These characteristics ensured comparability across facilities and strengthened the representativeness of the assessment within the regional hospital network. These characteristics ensured comparability across facilities based on key indicators such as level of care, service scope, catchment population, and organizational structure.

Within each hospital, 14 key informants were purposively selected, resulting in a total sample of 56 respondents across the four hospitals. The respondents included both clinical and administrative staff to obtain a range of views on how well the hospital was prepared for disasters.

The study’s inclusion of both clinical and administrative personnel enabled a comprehensive understanding of hospital preparedness, covering both operational service delivery and the managerial or coordination aspects of emergency and disaster management.

### Inclusion criteria

Participants were considered eligible for inclusion if they were employed at the selected hospitals during the study period and were actively working in emergency departments, hospital administration, planning units, or managerial roles. In addition, only those individuals who had a minimum of six months of work experience in their respective hospitals were included in the study. These criteria ensured that respondents possessed adequate familiarity with hospital systems, operational procedures, and emergency preparedness activities.

### Data collection tools

Data were collected using a structured questionnaire (see Appendix A) adapted from the WHO Hospital Safety Index (HSI) evaluation checklist. The HSI is a standardized test that checks how ready and safe a hospital is in case of an emergency or disaster.

The HSI framework consists of four assessment modules; however, this study utilized Module 4: Emergency and Disaster Management, which focuses specifically on institutional preparedness and response capacity. Further, Module 4 evaluates hospital preparedness across seven key domains, namely:

1. Coordination of emergency and disaster management activities.
2. Hospital emergency and disaster response and recovery planning.
3. Communication and information management.
4. Human resources management.
5. Logistics and financial resources.
6. Patient care and support services.
7. Evacuation, decontamination, and security.

The module includes 40 assessment indicators, each scored using a three-point Likert scale, where,

1 **=** Low level of preparedness.

2 = Moderate level of preparedness.

3 = High level of preparedness.

A score of “2” represents a moderate level of preparedness, indicating that the required structure or function exists but may not be fully developed or consistently implemented. Importantly, a rating of “low preparedness” (score = 1) does not necessarily indicate the absence of a structure (e.g., disaster committee or response plan), but rather reflects limited functionality, inadequate implementation, or insufficient capacity.

These indicators assess three key dimensions, namely availability, functionality, and effectiveness of preparedness mechanisms, including emergency coordination structures such as disaster response plans, staffing readiness, logistics systems, communication channels, and evacuation procedures. In addition to the HSI indicators, the questionnaire also included six demographic questions designed to capture respondents’ background characteristics, including gender, age, duty station, current position, professional experience, and highest level of education.

### Reliability of the survey instrument

The internal consistency of the HSI Module 4 assessment tool was evaluated using Cronbach’s alpha. The reliability analysis was performed on the overall 40-item disaster preparedness scale, which comprised indicators from all seven preparedness domains listed above. Cronbach’s alpha was calculated for the overall scale only; domain-specific Cronbach’s alpha coefficients were not computed. The overall Cronbach’s alpha coefficient was α = 0.734, indicating acceptable internal consistency and supporting the reliability of the instrument for assessing hospital emergency and disaster preparedness.

### Data collection procedure

Data collection was conducted through a self-administered questionnaire distributed by the principal researcher after receiving official permission from the Somali Regional Health Bureau. Each participating hospital designated a department head or focal person responsible for coordinating the survey within the institution. Along with the questionnaires, there was an official letter explaining the study’s purpose and a form for giving informed consent. Participants were given adequate time to complete the questionnaire, and completed forms were collected by the research team. The Chief Executive Officers (CEOs) of the hospitals were present during the data collection period to facilitate institutional coordination.

### Data management and analysis

All completed questionnaires were reviewed for completeness and consistency before data entry. No questionnaires were excluded from the analysis. The data were coded and entered into the IBM Statistical Package for the Social Sciences (SPSS) version 28.0 for analysis.

Descriptive statistical analysis was performed to summarize preparedness levels across hospitals. Statistical tests were used (t-test, ANOVA). For each of the 40 indicators in the WHO checklist, an average score was calculated based on respondents’ ratings. The mean scores were then converted into percentage preparedness scores to allow comparison across domains. Preparedness levels were classified according to the WHO Hospital Safety Index approach used in previous studies [4, 26].

- Low preparedness: ≤33.3%.
- Moderate preparedness: 33.4%–66.6%.
- High preparedness: >66.6%.

### Ethical approval

Ethical approval for the study was obtained from the Somali Regional Health Bureau and the institutional review boards of the participating hospitals. In addition, ethical clearance was granted by Maastricht University, where the research team is affiliated (approval number: FHML-REC/2023/125/Audit). The study was conducted in accordance with the ethical principles of the Declaration of Helsinki. Confidentiality and anonymity of participants were maintained throughout the study. All participants provided written informed consent before data collection and participation was voluntary. An information sheet explaining the objectives of the study was attached to the first page of the questionnaire, and the confidentiality and anonymity of respondents were ensured throughout the study.

## Results

### Participant characteristics

A total of 56 respondents participated in the study, with 14 respondents from each hospital. Most participants were male (76.8%) and aged 31–40 years (62.5%). More than half (62.5%**)** had 1–5 years of professional experience, while 28.6% had 5–10 years of experience; the largest proportions of respondents worked in the emergency unit (32.1%) and administration (26.8%). Other departments represented included wards, the Intensive Care Unit (ICU), the operating theater, pharmacy, laboratory, and outpatient services. The demographic characteristics of the study participants are presented in Table 1.

**Table 1:** Demographic characteristics

variables		No.	%
Hospitals	Degahbour	14	25%
	Godey	14	25%
	Karamara	14	25%
	Kabridahar	14	25%
Types of hospitals	General Public Hospital	4	100%
Respondents to disasters	Yes	56	100%
Gender	Female	13	23.20%
	Male	43	76.80%
Age	20–30	21	37.50%
	31–40	35	62.50%
Experience	Less than 1 year	5	8.90%
	1–5 years	35	62.50%
	5–10 years	16	28.60%
	Casualty	18	32.1
	Wards	6	10.7
Department	ICU	2	3.6
	Operating Theatre	4	7.1
	Pharmacy	3	5.4
	Laboratory	4	7.1
	Administration	15	26.8
	Outpatient Department/OPD	4	7.1

### Coordination of emergency and disaster management activities

The effectiveness of hospital emergency and disaster management largely depends on the presence of functional governance structures, clearly assigned roles, and operational coordination mechanisms. Table 2 summarizes the mean scores of key coordination indicators across the four hospitals.

All four hospitals reported having a hospital emergency and disaster committee. However, the mean score for this indicator was 1.5 ± 0.68, suggesting that these committees were either partially functional or existed largely in nominal form with limited operational capacity. Similarly, the indicator assessing committee member responsibilities and training received a low mean score (1.3 ± 0.54), indicating limited clarity of roles and insufficient training for committee members.

The availability of a designated emergency and disaster management coordinator was also limited, with a mean score of 1.2 ± 0.41, suggesting that most hospitals lacked clearly assigned leadership for disaster preparedness activities. Importantly, all information regarding the presence or absence of committees, plans, and coordination structures was obtained through self-reported responses from hospital key informants using the WHO HSI checklist, rather than direct observation of institutional documents. In addition, the preparedness program aimed at strengthening emergency response and recovery showed a very low score (1.1 ± 0.37), indicating minimal institutional efforts to systematically enhance preparedness capacity.

Although some hospitals reported a structure for incident management, the mean score of 1.7 ± 0.58 suggests that the system was only partially implemented, with limited staff assignments to specific incident management roles.

The lowest possible mean score (1.0 ± 0.00) for this indicator shows that none of the hospitals had a working Emergency Operations Center (EOC). Similarly, coordination with external actors remained limited. The mean score for coordination with local emergency and disaster management agencies was 1.2 ± 0.46, while coordination with the healthcare network scored slightly higher (1.5 ± 0.57), indicating weak inter-institutional collaboration mechanisms overall.

**Table 2:** Description of emergency and disaster preparedness of selected general public hospitals in Somali Region

Variables (Score Range = 1–3)	Mean ± SD
Coordination of emergency and disaster management activities [*N* = 56]	
Hospital emergency/disaster committee	1.5 ± 0.68
Committee member responsibilities and training	1.3 ± 0.54
Designated emergency and disaster management coordinator	1.2 ± 0.41
Preparedness program for strengthening emergency and disaster response and recovery	1.1 ± 0.37
Hospital incident management system	1.7 ± 0.58
Emergency Operations Center (EOC)	1.0 ± 0.00
Coordination mechanisms and cooperative arrangements with local emergency/disaster management agencies	1.2 ± 0.46
Coordination mechanisms and cooperative arrangements with the the healthcare network	1.5 ± 0.57
Hospital emergency and disaster response and recovery (*N* = 56)	
Hospital emergency or disaster response plan	1.1 ± 0.41
Hospital hazard-specific subplans	1.4 ± 0.60
Procedures to activate and deactivate plans	1.1 ± 0.44
Hospital emergency and disaster response plan exercises, evaluation and corrective actions	1.2 ± 0.48
Hospital recovery plan	1.2 ± 0.53
Communication and information management (*N* = 56)	
Emergency internal and external communication	1.2 ± 0.37
External stakeholder directory	1.2 ± 0.50
Procedures for communicating with the public and media	1.08 ± 0.28
Management of patient information	1.7 ± 0.68
Human resource HR (*N* = 56)	
Staff contact list	2.6 ± 0.58
Staff availability	1.3 ± 0.47
Mobilization and recruitment of personnel during an emergency or disaster	1.1 ± 0.33
Duties assigned to personnel for emergency or disaster response and recovery	1.1 ± 0.40
Well-being of hospital personnel during an emergency or disaster	1.2 ± 0.50
Logistics and finance (*N* = 56)	
Agreements with local suppliers and vendors for emergencies and disasters	1.3 ± 0.60
Transportation during an emergency	1.3 ± 0.64
Food and drinking-water during an emergency	1.3 ± 0.60
Financial resources. for emergencies and disasters	1.1 ± 0.40
Patient care and support services (*N* = 56)	
Continuity of emergency and critical care services	1.6 ± 0.69
Continuity of essential clinical support services	1.4 ± 0.60
Expansion of usable space for mass casualty incidents	1.4 ± 0.56
Triage for major emergencies and disasters	1.3 ± 0.60
Triage tags and other logistical supplies for mass casualty incidents	1.3 ± 0.55
System for referral, transfer and reception of patients	1.5 ± 0.63
Infection surveillance, prevention and control procedures	1.5 ± 0.68
Psychosocial services	1.3 ± 0.61
Post-mortem procedures in a mass fatality incident	1.3 ± 0.54
Evacuation, decontamination and security (*N* = 56)	
Evacuation plan	1.2 ± 0.42
Decontamination for chemical and radiological hazards	1.4 ± 0.60
Personal protection equipment and isolation for infectious diseases and epidemics	1.4 ± 0.62
Emergency security procedures	1.5 ± 0.63
Computer system network security	1.05 ± 0.22

### Emergency and disaster response and recovery

The level of emergency and disaster response and recovery of general public hospitals was the least prepared domain, scoring a total of 41.3% out of 100. Table 3 shows details of the parameters, the number of questions within each domain, score ranges, and hospital preparedness level. Consequently, all hospitals have an emergency and disaster response plan. However, there are no hazard-specific sub-plans, and the plans are not practiced using drills to assess and correct them as needed. Notably, all scores reflect the perceived level of preparedness based on Likert-scale responses (1 = low, 2 = moderate, 3 = high), not the absolute presence or absence of plans or systems.

**Table 3:** Level of emergency and disaster management preparedness of general public hospitals in the Somali region, Eastern Ethiopia, 2023

Parameters	Number of questions	Score ranges	Mean ± SD	Percent	Preparedness level
Preparedness level coordination of emergency and disaster management activities	8	8–24	10.7 ± 3.6	44.5%	Moderate
Hospital emergency and disaster response and recovery	5	5–15	6.2 ± 2.5	41.3%	Moderate
Communication and information management	4	4–12	5.3 ± 1.8	44.1%	Moderate
Human resources	5	5–15	7.4 ± 2.3	49.3%	Moderate
Logistics and finance	4	4–12	5.1 ± 2.2	42.5%	Moderate
Patient care and support services	9	9–27	12.8 ± 5.5	47.49%	Moderate
Evacuation, decontamination and security	5	5–15	6.6 ± 2.5	44%	Moderate
TOTAL	40	40–120	54.4 ± 20.4	45%	Moderate

### Communication and information management

Information is a necessary resource during an emergency or disaster. Planning, decision-making, and evaluating emergency health interventions are all based on the appropriate management and communication of information. Information is also essential for timely and efficient aid to individuals affected by emergencies or disasters. An important benefit of effective public and media communication is that it helps to mobilize the necessary funds for emergency medical assistance after a crisis. The study found that there is no organized way to inform the public and media during an emergency, but there is an external stakeholder directory for communication.

### Overall hospital disaster preparedness by hospitals

Table 4 presents the overall preparedness rating of four general public hospitals in the Somali Region. According to the HSI of the WHO scoring system, the achievement of an acceptable level of preparedness for a facility can occur with a score of only 66% and above, which is considered a high score. However, our findings indicate that all four hospitals fell below this desired preparedness level. Karamara General Hospital, for instance, received a 56.7%, scoring in some areas better at the level of emergency and disaster preparedness than other assessed hospitals, namely Degahbour (44.9%), Kabridahar (44.3%), and Godey (44.3%), demonstrating similar overall scores with slight variations. Further examination of specific subcategories reveals slight differences between the hospitals. As Karamara General Hospital leads the overall preparedness level, it also demonstrated strengths in certain areas when compared to other hospitals. Further, Karamara General Hospital, for instance, excelled in logistics and finance sections, scoring 5.6 out of 12. In contrast, Degahbour, Kabridahar, and Godey General hospitals scored 5.2, 5.07, and 4.6, respectively. Similarly, Karamara General Hospital outperformed other hospitals in terms of patient care and support, scoring 14 out of 27, whereas Degahbour, Kabridahar, and Godey scored 12.3, 12.5, and 12.4, respectively. However, with further scrutiny of the results, improvements are required in all hospitals for critical system supply, hospital recovery, hospital disaster plans, communication and information management, hospital decontamination, and business continuity.

The hospital capacity plays a crucial role in determining the average number of emergency patients who are admitted to each hospital on a regular business day. However, in the Ethiopian context, general public hospitals have additional responsibilities. They not only serve as referral centers for primary care but also function as training facilities for health officers, nurses, and emergency surgeons. Therefore, the general hospitals studied in the Somali Region can be compared based on factors such as their level of care, establishment history, population served, and the number of emergency beds allocated to them. Finally, in the domains of communication and information management, all hospitals scored almost similar points, ranging from 5.3 to 5.6, showing a moderate level of preparedness. However, all findings reflect perceived preparedness levels measured using the WHO HSI Likert-scale tool, rather than verification of document existence or institutional records.

**Table 4:** Assessed general public hospitals’ overall preparedness rating

Module 4: Emergency and disaster management	Total Criteria	Score Ranges	Karamara Hospital	Degahbour Hospital	Kabridahar Hospital	Godey Hospital
4.1 Coordination of emergency and disaster management Activities	8	8–24	10.6	10.6	10.5	11.3
4.2 Hospital emergency and disaster response and recovery	5	5–15	6.4	6	5.7	6.6
4.3 Communication and information management	4	4–12	5.6	5.6	5.4	5.3
4.4 Human resource/HR	5	5–15	7.6	7.6	7.3	6.9
4.5 Logistics and finance	4	4–12	5.6	5.2	5.07	4.6
4.6 Patient care and support services	9	9–27	14	12.3	12.5	12.4
4.7 Evacuation, decontamination and security	5	5–15	6.9	6.6	6.7	6.1
Total module score per hospital	40	40–120	56.7	53.9	53.17	53.2
Overall performance level per hospital (%)			**47.2%**	**44.9%**	**44.3%**	**44.3%**

### Comparative analyses by respondent characteristics

Comparative analyses were conducted to examine differences in total preparedness scores by gender, years of professional experience, department, and hospital. Independent samples t-tests and one-way ANOVA were applied as appropriate. The results showed no statistically significant differences in preparedness scores across gender (*p* = 0.63), years of experience (*p* = 0.48), department (*p* = 0.39), or hospital (*p* = 0.41). Although Karamara Hospital demonstrated a slightly higher mean preparedness score (56.7 ± 18.7) compared with the other hospitals, the differences were not statistically significant. The results of the subgroup comparisons are presented in Table 5.

**Table 5:** Template for subgroup comparison of total preparedness scores

Variable	Category	*n*	Mean total preparedness score ± SD	Statistical test	*p*-value
Gender	Male	43	54.8 ± 19.8	Independent samples t-test	0.63
	Female	13	53.1 ± 21.2		
Years of experience	< 1 year	5	51.6 ± 18.5	One-way ANOVA	0.48
	1–5 years	35	53.7 ± 20.6		
	5–10 years	16	56.2 ± 19.3		
Department	Casualty/Emergency	18	55.6 ± 19.7	One-way ANOVA	0.39
	Administration	15	52.9 ± 21.5		
	Other departments	23	54.3 ± 20.1		
Hospitals	Degahbour	14	53.9 ± 19.9	One-way ANOVA	0.41
	Gode	14	53.2 ± 21.0		
	Karamara	14	56.7 ± 18.7		
	Kabridahar	14	53.2 ± 21.4		

## Discussion

This study assessed the level of emergency and disaster preparedness in four zonal hospitals in the Somali Regional State of Ethiopia using the WHO Hospital Safety Index emergency and disaster management module. The findings show that the overall preparedness level of the assessed hospitals was 45%, indicating a moderate level of disaster preparedness. Among the assessed domains, hospital emergency, disaster response, recovery, logistics, and finance demonstrated the lowest preparedness levels, while human resources showed relatively higher preparedness, although it remained within the moderate preparedness category. These findings indicate that hospitals in the Somali Region presently possess insufficient institutional capacity to adequately address large-scale emergencies and disasters.

The findings of this study are consistent with previous research conducted in different regions of Ethiopia, including studies from northwest, southwest, and western Ethiopia, as well as Addis Ababa, which reported moderate levels of hospital disaster preparedness [4, 16, 25–29]. Research in Tanzania and Saudi Arabia has yielded analogous results, indicating hospitals’ inadequate preparedness for disaster scenarios [11, 42]. However, preparedness levels reported in this study appear higher than those reported in some Kenyan hospitals [5, 43]. Variations in health system resources, training opportunities, and institutional awareness of disaster risk across settings may explain these differences.

A key finding of this study is the limited functionality of disaster management systems in hospitals. Although all assessed hospitals reported the existence of emergency or disaster committees, the results suggest that these committees are largely inactive and lack clearly defined responsibilities. Similarly, diagrams often depicted incident management systems without assigning any operational roles. These findings are consistent with previous studies conducted in western Ethiopia and Indonesia, where formal disaster management systems in hospitals existed but were not fully operational [27, 44].

The absence of functional emergency operations centers and regular emergency drills further highlights institutional preparedness weaknesses. Regular simulation exercises and clearly defined incident command structures are essential components of effective disaster management systems [45]. Strengthening coordination mechanisms within hospitals would therefore improve decision-making and response capacity during emergencies.

Human resources play a central role in disaster response, as healthcare workers are responsible for delivering emergency care and coordinating response activities [46, 47]. In this study, the human resources domain showed relatively higher preparedness compared with other domains, mainly due to the presence of staff contact lists. However, several critical elements were still lacking, including staff mobilization mechanisms, clearly defined emergency roles, and staff training programs.

These findings diverge from studies conducted in other settings that implemented training programs for disaster preparedness more systematically [48, 49]. This suggests that hospitals in the Somali Region may benefit from regular disaster preparedness training, role clarification, and staff capacity-building initiatives to strengthen emergency response readiness.

This study found that logistics and financial readiness were two of the weakest areas. The results indicate that hospitals lack formal agreements with suppliers for emergency supplies and resources. Disaster situations lack sufficient planning for essentials like transportation, food, drinking water, and emergency medical equipment. Research in northwest Ethiopia and other low-income areas has yielded comparable results [4, 50].

Limited financial resources and logistical capacity remain major challenges for disaster preparedness in many low- and middle-income countries [51]. In resource-constrained settings such as the Somali Region, establishing partnerships with local suppliers and developing contingency funding mechanisms could significantly improve preparedness for emergency response. The findings also highlight important regional disparities in disaster preparedness, as hospitals in the Somali Region face additional challenges related to geographic remoteness, limited infrastructure, and constrained supply-chain systems. These contextual factors may further hinder the timely mobilization of emergency resources compared with better-resourced regions of Ethiopia [52].

Effective communication systems are essential for coordinating disaster response, sharing information, and mobilizing resources. The findings of this study indicate that communication and information management systems in the assessed hospitals remain weak, particularly with regard to communication with external stakeholders and the public. These findings are consistent with previous studies conducted in Tanzania and other low-income settings that reported insufficient communication infrastructure in hospitals [11, 51]. However, studies conducted in high-income countries such as Italy have reported more advanced communication systems, highlighting disparities in institutional preparedness between health systems with different levels of resources [53]. The observed communication gaps may reflect broader regional constraints, including limited information technology infrastructure, inadequate emergency communication networks, and shortages of trained personnel responsible for emergency coordination and information management.

The study found that hospitals had some capacity to maintain essential clinical services during emergencies, including triage systems and referral mechanisms. However, the provision of psychosocial support services for disaster victims was largely absent. This finding is consistent with studies conducted in Ethiopia and Tanzania that reported similar gaps in mental health and psychosocial support during disaster response [11, 26, 54]. Integrating psychosocial support services into hospital plans for disaster preparedness would, therefore, strengthen patient-centered emergency response systems. Furthermore, evidence regarding the integration of psychosocial support into hospital disaster preparedness remains limited in pastoralist and humanitarian settings such as the Somali Region, highlighting an important knowledge gap that warrants further investigation.

The findings also indicate weaknesses in hospital evacuation procedures, decontamination capacity, and security systems. Despite the availability of personal protective equipment in some hospitals, there were limited comprehensive plans for evacuation and hazard-specific decontamination. Several studies assessing hospital disaster preparedness in low-resource settings have reported similar challenges [12, 26, 51]. This study contributes new evidence on these preparedness domains in the Somali Region, where empirical data on hospital disaster readiness have previously been scarce. By documenting deficiencies in evacuation planning, decontamination capacity, and security systems, the study helps address an important regional knowledge gap and provides evidence to inform future preparedness interventions and policy decisions.

## Limitations of the study

This study has several limitations that should be considered when interpreting the findings. First, the study was conducted in only four general public hospitals, which may limit the generalizability of the results to other hospitals in Ethiopia. Second, the study employed purposive sampling, which may introduce selection bias. Third, the sample showed imbalances in gender and age distribution, as the majority of respondents were male and within a limited age range. Fourth, the study relied on self-reported survey data, which may be influenced by social desirability bias, as respondents may have over- or under-reported preparedness practices. Finally, the cross-sectional design captures preparedness at a single point and may not reflect changes in disaster preparedness over time.

As a result, the findings may not capture temporal variations in preparedness levels arising from changes in staffing, training activities, resource availability, policy implementation, or recent emergency events. In addition, preparedness capacities may fluctuate over time due to seasonal hazards, disease outbreaks, or institutional improvements, which could not be assessed within the study period. Therefore, the results should be interpreted as a snapshot of preparedness status rather than an assessment of long-term trends or changes in preparedness over time.

Despite these limitations, the study provides important empirical evidence on hospital disaster preparedness in the Somali Regional State, where limited research has previously been conducted.

## Conclusion

This study’s results showed that the emergency and disaster preparedness levels in general public hospitals in the Somali Region, Ethiopia, have suboptimal emergency and disaster preparedness, with an overall preparedness score of 45%. Important discrepancies were found within major preparedness areas such as emergency response plans, preparedness for logistical and financial issues, communication systems, and disaster management mechanisms. Based on the high vulnerability of the region to disasters and public health emergency events, inadequate preparedness may further impair the capacity of the hospitals to tackle disasters and public health emergencies when they happen. Institutional disaster preparedness needs to be addressed by means of improving emergency response planning, coordinating mechanisms, capacity building, mobilization of staff and resources, and communication system.

## Appendices

### Appendix A

WHO Hospital Safety Index Module 4.

### Questionnaire

Dear Colleague,

My name is Bashir Farah, and I am a PhD candidate at Maastricht University in the Netherlands. As part of my PhD study in health services research, I am required to conduct research. The topic for my research is hospital disaster preparedness in the Somali regional state of Ethiopia. A study of Karamara, Degahbour, Gode, and Kabridahar General Hospitals for crisis management. This questionnaire was developed for one of my studies and has pure academic purposes.

I am kindly asking you to be a participant in the study. If you agree, you are kindly requested to complete the questionnaire below as accurately and completely as possible. The objective of the study is to assess your knowledge, attitudes, and practices with regard to the management of emergencies and disasters (for example, floods, drought, and road traffic accidents). The study has been approved by the management of General Hospitals and the Somali Regional Health Bureau.

The information you give will be kept confidential, and your name shall not appear on the questionnaire and will not keep records of any information that can identify you. Participation in the study is voluntary, and you are under no obligation to fill out the questionnaire. In addition to the fulfillment of my studies, the information gathered will be used for the provision of better emergency services not only at selected general public hospitals in the specific provinces but also at other hospitals in the Somali region. By completing the questionnaire, you also consent to participate in the study.

To complete the questionnaire, kindly indicate your answer with a cross (**X**) in the appropriate block or write in the space provided.

Warmest regards,

Bashir Farah.

## Instructions

The questionnaire is organized into two sections. Section one covers demographics-related questions whereas section two considers the level of preparedness of a hospital’s organization, personnel and essential operations to provide patient services in response to an emergency or disaster.

This checklist is for assessing the level of disaster and emergency preparedness of Karamara, Degahbour, Gode, and Kabridahar General Hospitals in the Somali Regional State, Ethiopia.

1. Indicate with an (X) in the relevant column showing whether the component is available or not.
2. Write your comment in the space provided.
3. Where possible, conduct a physical check of the hospital to find out if the listed component or facility is available and make comments in the space provided.

## Section  1: Demographics

**Table:** 

What is your gender?					
1. Male				2. Female	

**Table:** 

	Age at next birthday				
1. Below 20		2. 20–30		3. 31–40	
4. 41–50		5. 51–60		6. 61+	

**Table:** 

	Indicate your duty station				
1. Casualty		2. Wards		3. Intensive Care Unit (ICU)	
4. Theatre		5. Pharmacy		6. Laboratory	
7. Administration		8. Outpatients Department (OPD)			
9. Other (specify)					

**Table:** 

	What is your current position?				
1. Medical Officer		2. Registered Nurse		3. Pharmacist	
4. Laboratory		5. Enrolled Nurse			
6. Specialist (specify)					
7. Administrator (specify)					
8. Other (specify)					

**Table:** 

	How many years have you worked in your current position?				
1. Less than 1 year		2. 1–5 years		3. 5–10 years	
4. 10–15 years		5. 15–20		6. More than 20 years	

**Table:** 

6.	What is your highest level of education?				
1. No schooling		2. Primary school		3. Secondary school	
4. Certificate		5. Diploma		6. Undergraduate	
7. Postgraduate					
8. Other (specify)					

## Section 2: Emergency and disaster management

**Table:** 

4.1 Coordination of emergency and disaster management activities	Level			Observations Evaluators’ comments
	Low	Average	High	
**1. Hospital Emergency/Disaster Committee** Safety ratings: Low = Committee does not exist, or 1–3 departments or disciplines represented; Average = Committee exists with 4–5 departments or disciplines represented, but is not fulfilling functions effectively; High = Committee exists with 6 or more departments or disciplines represented and is fulfilling functions effectively				
**2. Committee member responsibilities and training** Safety ratings: Low = Committee does not exist or members are untrained and responsibilities not assigned; Average = Members have received training and have been officially assigned; High = All members are trained and are actively fulfilling their roles and responsibilities.				
**3 Designated emergency and disaster management coordinator** Safety ratings: Low = There is no staff member who has been assigned responsibilities as the emergency/disaster management coordinator; Average = Emergency/disaster management coordination tasks have been assigned to a staff member, but it is not his/her main task; High = A staff member is assigned the emergency and disaster management coordination responsibilities as his/her main task, is fulfilling the role of implementing the a hospital’s preparedness Programme.				
**4 Preparedness Programme for strengthening emergency and disaster response and recovery** Safety ratings: Low = A Programme for strengthening preparedness, response and recovery does not exist or, if it exists, no preparedness activities are being implemented; Average = A programme for strengthening preparedness, response and recovery exists and some activities are being implemented; High = A programme for strengthening preparedness, response and recovery is being fully implemented under the leadership of the Hospital Emergency/Disaster Committee.				
**5. Hospital incident management system**	**Level**			**Observations** Evaluator’s comments
	**Low**	**Average**	**High**	
Safety ratings: Low = No arrangements for hospital incident management exist; Average = Staff assigned to key hospital incident management positions but with no written procedures to operationalize its functions; High = Hospital incident management procedures exist and are fully operational with properly trained personnel to assume different coordination roles and responsibilities.				
**6. Emergency Operations Centre (EOC)** Safety ratings: Low = The EOC is not designated or is in an unsafe or insecure location; Average = The designated EOC is in a safe, secure and accessible location, but would have limited operational capacity immediately in an emergency; High = The EOC is in a safe, secure, and accessible location with immediate operational capacity.				
**7. Coordination mechanisms and cooperative arrangements with local emergency/disaster management agencies** Safety ratings: Low = No arrangements exist; Average = Arrangements exist but are not fully operational; High = Arrangements exist and are fully operational.				
**8. Coordination mechanisms and cooperative arrangements with the healthcare network** Safety ratings: Low = No arrangements exist; Average = Arrangements exist but are not fully operational; High = Arrangements exist and are fully operational				

**Table:** 

4.2 Hospital emergency and disaster response and recovery planning	Level			Observations Evaluators’ comments
	Low	Average	High	
**1. Hospital emergency or disaster response plan** Safety ratings: Low = Plan is not documented; Average = Documented plan is complete, but is not easily accessible, not up to date (more than 12 months since the last update); High = Plan is complete, easily accessible, reviewed/updated at least annually, and resources are available to implement the plan.				
**2. Hospital hazard-specific subplans** Safety ratings: Low = Hazard-specific response subplans are not documented; Average = Documented plans are complete but not easily accessible, not up to date (more than 12 months since last review/update); High = Documented plans are complete, reviewed/updated at least annually, and resources are available to implement the plans.				
**3. Procedures to activate and deactivate plans** Safety ratings: Low = Procedures do not exist or exist only as a document; Average = Procedures exist, personnel have been trained, but procedures are not updated or tested annually; High = Up-to-date procedures exist, personnel have been trained, and procedures have been tested at least annually.				
**4. Hospital emergency and disaster response plan exercises**,** evaluation and corrective actions** Safety ratings: Low = Response plan and subplans have not been tested; Average = Response plan or subplans are tested, but are not tested at least annually; High = Response plan or subplans are tested at least annually and updated according to the exercise results.				
**5. Hospital recovery plan** Safety ratings: Low = Recovery plan is not documented; Average = Documented plan is complete, but not easily accessible, not up-to-date (more than 12 months since last review/update); High = Documented plan is complete, easily accessible, and reviewed/updated at least annually				

**Table:** 

4.3 Communication and information management	Level			Observations Evaluators’ comments
	Low	Average	High	
**1. Emergency internal and external communication** Safety ratings: Low = Central internal and external communication system functions inconsistently or incompletely; operators are not trained in emergency communication; Average = System functions appropriately, operators have received some training in emergency communication, tests are not conducted at least annually; High = System functions completely and operators are fully trained in emergency use, and tests of the system are conducted at least annually.				
**2. External stakeholder directory** Safety ratings: Low = Directory of external stakeholders does not exist; Average = Directory exists but is not current (more than 3 months since it was updated); High = Directory is available, is up to date and is held by key emergency response staff.				
**3. Procedures for communicating with the public and media** Safety ratings: Low = Procedures do not exist, no spokesperson nominated; Average = Procedures exist and nominated spokespersons have been trained; High = Procedures exist, nominated spokespersons have been trained, and procedures have been tested at least annually.				
**4. Management of patient information** Safety ratings: Low = Procedures for emergency situations. do not exist; Average = Procedures for emergency situations exist and personnel have been trained but no resources are available. High = Procedures for emergency situations exist, personnel have been trained, and resources are in place for implementation.				

**Table:** 

4.4 Human resources	Level			Observations Evaluators’ comments	
	Low	Average	High		
**1. Staff contact list** Safety ratings: Low = Contact list does not exist; Average = List exists, but is not current (more than 3 months since it was updated); High = List is available and up to date.					
**2. Staff availability** Safety ratings: Low = Less than 50% of staff are available to run each department adequately; Average = 50–80% of staff are available; High = 80–100% of staff are available.					
**3. Mobilization and recruitment of personnel during an emergency** **or disaster** Safety ratings: Low = Procedures do not exist or exist only in a document; Average = Procedures exist, and personnel have been trained, but the human resources for an emergency situation are not available; High = Procedures exist, personnel have been trained, and the human resources are available to meet anticipated needs in an emergency.					
**4. Duties assigned to personnel for emergency or disaster response** **and recovery** Safety ratings: Low = Emergency assignments do not exist or are not documented; Average = Duties are identified, some (but not all) personnel receive written assignments or training; High = Written duties are assigned, and training or an exercise is conducted for all personnel at least annually.					
**5. Well-being of hospital personnel during an emergency or disaster** Safety ratings: Low = A designated space and measures do not exist; Average = Space has been designated, but measures cover less than 72 h; High = Measures are ensured for at least 72 h.					

**Table:** 

4.5 Logistics and finance	Level			Observations Evaluators’ comments
	Low	Average	High	
**1. Agreements with local suppliers and vendors for emergencies and** **disasters** Safety ratings: Low = No arrangements exist; Average = Arrangements exist, but are not fully operational; High = Arrangements exist and are fully operational.				
**2. Transportation during an emergency** Safety ratings: Low = Ambulances and other vehicles and modes of transportation are not available; Average = Some vehicles are available, but not in sufficient numbers for a major emergency or disaster; High = Appropriate vehicles in sufficient numbers are available during emergencies/disasters.				
**3. Food and drinking-water during an emergency** Safety ratings: Low = Procedures for food and drinking-water for emergencies arenonexistentt; Average = Procedures exist, food and drinking water is guaranteed for less than 72 h; High = Food and drinking water for emergencies is guaranteed for at least 72 h.				
**4. Financial resources for emergencies and disasters** Safety ratings: Low = Emergency budget or mechanism to access emergency funds is not in place; Average = Funds are budgeted and mechanisms are available but cover less than 72 h; High = Sufficient funds are guaranteed for 72 h or more				

**Table:** 

4.6 Patient care and support services	Level			Observations Evaluators’ comments
	Low	Average	High	
**1. Continuity of emergency and critical care services** Safety ratings: Low = Procedures do not exist or exist only as a document; Average = Procedures exist, personnel have been trained but would not be available at all times; High = Procedures exist, personnel have been trained, and resources are available to implement procedures at maximum hospital capacity for emergency and disaster situations at all times.				
**2. Continuity of essential clinical support services** Safety ratings: Low = Procedures do not exist or exist only as a document; Average = Procedures exist and personnel have been trained but would not be available at all times; High = Procedures exists, personnel have been trained, and resources are available to implement procedures at maximum hospital capacity for emergency and disaster situations at all times.				
**3. Expansion of usable space for mass casualty incidents** Safety ratings: Low = Space for expansion has not been identified; Average = Space has been identified; equipment, supplies and procedures are available to carry out the expansion and staff have been trained, but testing has not been conducted; High = Procedures exist and have been tested, personnel have been trained, and equipment, supplies and other resources are available to carry out the expansion of space.				
**4. Triage for major emergencies and disasters** Safety ratings: Low = Designated triage location or procedures do not exist; Average = Triage location and procedures exist and personnel have been trained, but procedures have not been tested for emergency and disaster situations; High = Location and procedures exist and have been tested, personnel have been trained, and resources are in place to implement at maximum hospital capacity in emergency and disaster situations.				
**5. Triage tags and other logistical supplies for mass casualty incidents** Safety ratings: Low = Nonexistent; Average = Supply covers less than 72 h of maximum hospital capacity; High = Supply guaranteed for at least 72 h of maximum hospital capacity.				
**6. System for referral**,** transfer and reception of patients** Safety ratings: Low = Procedures do not exist or exist only as a document; Average = Procedures exist and personnel have been trained, but procedures have not been tested for emergency or disaster situations; High = Procedures exist and have been tested, personnel have been trained, and resources are available to implement measures at maximum hospital capacity in emergency or disaster situations.				
**7. Infection surveillance**,** prevention and control procedures**	**Level**			**Observations** Evaluator’s comments
	**Low**	**Average**	**High**	
Safety ratings: Low = Policies and procedures do not exist; standard precautions for infection prevention and control are not followed routinely; Average = Policies and procedures exist, standard precautions are routinely followed, personnel have been trained, but the level of resources required for emergency and disaster situations, including epidemics, is not available; High = Policies and procedures exist, infection prevention and control measures are in place, personnel have been trained, and resources are available to implement measures at maximum hospital capacity in emergency and disaster situations.				
**8. Psychosocial services** Safety ratings: Low = Procedures do not exist or exist only as a document; Average = Procedures exist and personnel have been trained, but the level of resources required for emergency and disaster situations is not available; High = Procedures exist, personnel have been trained, and resources are available for implementation of procedures at maximum hospital capacity in emergency and disaster situations.				
**9. Post-mortem procedures in a mass fatality incident** Safety ratings: Low = Procedures for a mass fatality incident do not exist or exist only as a document; Average = Procedures exist and personnel have been trained, but the level of resources required for emergency and disaster situations is not available; High = Procedures exist, personnel have been trained, and resources are available for implementation of procedures at maximum hospital capacity in emergency and disaster situations				

**Table:** 

4.7 Evacuation, decontamination and security	Level			Observations Evaluators’ comments
	Low	Average	High	
**1. Evacuation plan** Safety ratings: Low = Plan does not exist or exists only as a document; Average =plann exists and personnel have been trained in procedures, but tests are not conducted regularly; High = Plan exists, personnel have been trained, and evacuation drills are held at least annually.				
**2. Decontamination for chemical and radiological hazards** Safety ratings: Low = No personal protective equipment is available for immediate use by hospital staff, or no decontamination area exists; Average = Personal protective equipment is available for immediate use, decontamination areas are established, staff training and drills are not conducted annually; High = Personal protective equipment is available for immediate use, decontamination areas are established and personnel are trained and tested at least annually.				
**3. Personal protection equipment and isolation for infectious diseases and epidemics** Safety ratings: Low = No personal protective equipment is available for immediate use by hospital staff, or no isolation area exists; Average = Supply is available for immediate use, but is sufficient for less than 72 h of maximum hospital capacity, isolation areas are established, staff training and testing of procedures are not conducted annually; High = Supply is guaranteed for at least 72 h of maximum hospital capacity and alternate sources are in place for resupply, isolation areas are established, staff training and testing of procedures are conducted at least annually.				
**4. Emergency security procedures** Safety ratings: Low = Emergency security procedures do not exist or exist only as a document; Average = Documented procedures exist and personnel have been trained in emergency security procedures but testing is not conducted at least annually; High = Personnel are trained and tests of the documented procedures are held at least annually.				

## Data Availability

The datasets generated and analyzed during the current study are not publicly available owing to institutional and ethical requirements protecting participant confidentiality. Data are, however, available upon reasonable request accessed from the corresponding author.

## Abbreviations

CEO: Chief Executive Officers
EOC: Emergency Operation Center
LMICs: Low-and middle -income countries
ICU: Intensive Care Unit
WHO: World Health Organization
HIS: Hospital Safety Index
SSA: Sub-Saharan Africa
PAHO: Pan American Health Organization
SRS: Somali Regional State
UN: United Nations
SPSS: Statistical Package for the Social Sciences
SRS: Somali Regional State
